# Chasing the hare - Evaluating the phylogenetic utility of a nuclear single copy gene region at and below species level within the species rich group *Peperomia *(Piperaceae)

**DOI:** 10.1186/1471-2148-11-357

**Published:** 2011-12-12

**Authors:** Julia Naumann, Lars Symmank, Marie-Stéphanie Samain, Kai F Müller, Christoph Neinhuis, Claude W dePamphilis, Stefan Wanke

**Affiliations:** 1 Technische Universität Dresden, Institut für Botanik, D-01062 Dresden, Germany; 2 Ghent University, Department of Biology, Research Group Spermatophytes, B-9000 Gent, Belgium; 3 University of Münster, Institute for Evolution and Biodiversity, D-48149 Münster, Germany; 4 Department of Biology, The Pennsylvania State University, University Park, Pennsylvania 16802, USA

## Abstract

**Background:**

The rapidly increasing number of available plant genomes opens up almost unlimited prospects for biology in general and molecular phylogenetics in particular. A recent study took advantage of this data and identified a set of nuclear genes that occur in single copy in multiple sequenced angiosperms. The present study is the first to apply genomic sequence of one of these low copy genes, *agt1*, as a phylogenetic marker for species-level phylogenetics. Its utility is compared to the performance of several coding and non-coding chloroplast loci that have been suggested as most applicable for this taxonomic level. As a model group, we chose *Tildenia*, a subgenus of *Peperomia *(Piperaceae), one of the largest plant genera. Relationships are particularly difficult to resolve within these species rich groups due to low levels of polymorphisms and fast or recent radiation. Therefore, *Tildenia *is a perfect test case for applying new phylogenetic tools.

**Results:**

We show that the nuclear marker *agt1*, and in particular the *agt1 *introns, provide a significantly increased phylogenetic signal compared to chloroplast markers commonly used for low level phylogenetics. 25% of aligned characters from *agt1 *intron sequence are parsimony informative. In comparison, the introns and spacer of several common chloroplast markers (*trnK *intron, *trnK*-*psbA *spacer, *ndhF*-*rpl32 *spacer, *rpl32*-*trnL *spacer, *psbA*-*trnH *spacer) provide less than 10% parsimony informative characters. The *agt1 *dataset provides a deeper resolution than the chloroplast markers in *Tildenia*.

**Conclusions:**

Single (or very low) copy nuclear genes are of immense value in plant phylogenetics. Compared to other nuclear genes that are members of gene families of all sizes, lab effort, such as cloning, can be kept to a minimum. They also provide regions with different phylogenetic content deriving from coding and non-coding parts of different length. Thus, they can be applied to a wide range of taxonomic levels from family down to population level. As more plant genomes are sequenced, we will obtain increasingly precise information about which genes return to single copy most rapidly following gene duplication and may be most useful across a wide range of plant groups.

## Background

Molecular phylogenetics has made remarkable progress in recent decades toward the reconstruction of a largely resolved 'tree of life' [1]. In flowering plants, the relationships are now clear from the deepest branches through to the family level, with only a few exceptions [2-4]. However, developing strong phylogenetic hypotheses for more closely related plants at the interface of species level and population level has been, and still is, quite challenging. At this level, gene exchange and recombination is still possible, and low sequence divergence often limits phylogenetic resolution. As a result, multiple independent phylogenetic markers with sufficient sequence divergence are necessary, and analytical approaches from population genetics may in some cases be more appropriate than traditional phylogenetic methods [5,6].

The most commonly used marker regions in plant phylogenetics are coding and non-coding sequences from the chloroplast genome and ribosomal gene regions located in the nucleus. Recently, whole plastid genomes (plastomes) have been used to address deep branching questions [e.g. [7-11]] in plants, and a rapid increase in plastome scale data is underway. Mitochondrial genes are not as widely used because of typically very slow sequence evolution and some complexities such as extreme rate variation in some lineages and occurrences of horizontal gene transfer [12,13], gene conversion and processed paralogy [e.g. [14]]. Nevertheless, these genes do play a substantial role for phylogenies of for instance parasitic plants [e.g. [13,15]] where plastid genomes can be highly modified [16,17]. However, some of the most extensive efforts to date to resolve the "deep branches" in flowering plant phylogenetics have involved large taxon samples with sequence data obtained from up to 17 genes representing all three genomic compartments [18].

"The Tortoise and the Hare" series [19-21] addressed the utility of nuclear and chloroplast loci for low level phylogenetics. These authors noticed an increased resolution for recent branching events with the nuclear gene *Adh*, but the chloroplast markers required much less laboratory effort. Subsequently, a number of promising chloroplast regions have been used to address questions at low taxonomic levels [21]. Plastomes provide numerous genes, introns, and intergenic regions; amplification and direct sequencing of PCR product is easy as a single cell harbors 1000 or more plastids with multiple (essentially) identical plastid genomes. Plastomes provide an almost unmatched source of orthologous sequence that is not complicated by gene family duplication, and multiple genes can be concatenated to provide a very long sample of orthologous sequence. However, plastome markers are tightly linked on a single (typically) non-recombining molecule that usually reflects only the maternal lineage in angiosperms, limiting the generality of plastid DNA as the sole source of evolutionary markers at and below species level. In addition, plastomes are limited in their variability (i.e. substitution rates, parsimony informative sites) and therefore their utility for low taxonomic level studies may be restricted [19,22].

The intergenic spacer (ITS) regions of nrDNA often provide higher variability, but their orthology usually remains an assumption not tested prior to their use in phylogenetic analysis. Hundreds to thousands of copies of highly conserved nrDNA gene regions located in the nucleus make those markers easy to amplify, but their evolution by tandem duplication events results in a gene array more accurately described as a gene family or large collection of paralogs [23,24]. The paralogous nature of nrDNA can complicate the reconstruction of phylogenetic relationships, as it is impossible to determine orthologous copies from such a large copy number. As a consequence, divergent paralogs may be inadvertently sampled from different organisms in a study; when included in phylogenetic reconstruction, artifacts can appear [e.g. [25]].

Nuclear markers (excluding nrDNA marker) are essential for a broad range of evolutionary investigation, including systematics and character evolution [e.g. [26]], hybridization [e.g. [27]], polyploidization [e.g. [28]], biogeography [29], origins of domestication [e.g. [30]], and speciation [e.g. [31]]. Occurring independently all over the nuclear genome in a virtually inexhaustible repertoire in terms of both number and variability, bi-parentally inherited nuclear loci are promising on different levels, especially when compared to organellar markers.

The first attempts to establish nuclear markers other than ribosomal genes yielded a substantial number of low copy nuclear genes (LCNG) [32]. LCNG, such as the *ADH-*genes [33], *pistillata *[34], *GPAT *[35], *PRK *[36] or *LEAFY *[37] were applied in numerous studies. These genes are known to occur either in single copy in one or more focal species in the study group, or as members of small gene families [38] and may not occur in any specific plant lineage. Complete sampling of gene families often involves intense experimental efforts to clone and sequence all members of a family. Thus, for practical reasons, single loci are mostly preferred.

Many different approaches have been used to identify useful nuclear single copy loci in plants, with results varying widely in terms of both the general idea and computational effort. In recent years, the advent of next generation sequencing technologies (NGS), bioinformatic progress, and publicly available sequence data have greatly facilitated the identification of such loci [e.g. [39]]. These rich sources of sequence information have been utilized by many research groups, who identified new nuclear markers for plant, animal and fungal phylogenetics to reveal relationships that could not be resolved with organelle or nuclear ribosomal DNA markers [22,40-47]. All of them focus on genes that occur in the nucleus in single copy in a sample of organisms with sequenced genomes. The nucleus provides a vast repertoire of unlinked markers. However, since there are thousands of genes in the nucleus, marker selection is challenging and the majority of nuclear genes occur in small to large gene families. The present study is based on the approach published in Duarte *et al*. [22], where a global classification of plant protein coding sequences (Tribes) was used to identify a collection of 959 genes that are represented by exactly one copy in each of four sequenced angiosperm genomes (*Arabidopsis*, *Populus*, *Vitis*, and *Oryza*). "Tribes" are collections of related genes in a specified set of genomes produced by the gene clustering program MCL-Tribe [39,48]. While many PlantTribes approximate gene families [39], the global classification also identifies genes that are members of highly distinctive clusters that lack closely related paralogs, including clusters with only a single gene in each taxon. The set of genes identified in the analysis was called APVO SSCG (*Arabidopsis*, *Populus*, *Vitis*, *Oryza *shared single copy genes). The *agt1 *gene, applied in the present study, is part of that set and the abbreviation nSCG (nuclear single copy gene) that is used here refers to this approach. We use the term nSCG operationally, referring to a gene that has been identified as single copy in global gene classification of a specified set of genomes, here APVO. This does not necessarily mean that the gene will be single copy in any given lineage, particularly in very recent polyploids [22,49], where the entire gene set has been recently duplicated, and the process of duplicate gene loss is underway. However, as larger numbers of genomes are interrogated, genes that continue to be found in single copy in all but the most recent polyploids are more likely to be single copy in a given uncharacterized lineage.

Hughes *et al*. [50] proposed the exploration of nuclear loci other than nrDNA for phylogenetic reconstruction, which would require the abandonment of 'universal thinking'. The enhanced variability of nuclear loci compared to other markers provides great potential, but limits the likelihood of identifying universal amplification primers that will function across a wide taxonomic range. In fact, it might be difficult to identify universal gene loci because polyploidy is prevalent and frequent in plants. Duplicated genes are often lost by both random and selective processes in a short time, but that also means there is a chance to encounter multiple gene copies in a specific lineage [22].

*Peperomia *(Piperaceae) ranks among the ten largest angiosperm genera, with approximately 1,650 species [51]. The phylogenetics and classification of such species-rich clades has long been very challenging. In addition, morphological characters have been shown to be subject to parallel evolution and extreme reduction, resulting in a paucity of synapomorphies [52,53]. Moreover, speciation within *Peperomia *has likely happened comparatively recently [51], and as a consequence, reconstructed phylogenies often lack resolution at the species and population level [54]. Recent backbone phylogenies were based on over 4000 molecular characters to overcome the lack of variability [52,53]. Therefore, *Peperomia *is an ideal candidate for testing the performance of a nSCG region and comparing outcomes with variable chloroplast markers such as those suggested by Shaw *et al*. [20,21]. Within this study, we focus on closely related species belonging to *Peperomia *subgenus *Tildenia *where currently 59 species are recognized [55,56].

## Results

The *agt1 *gene studied here is an ortholog of At2G13360 in *Arabidopsis thaliana*, where it is known to catalyze the Alanine:Glyoxylate Aminotransferase reaction located in peroxisomes [57]. It was identified to be shared between four angiosperm genomes (*Arabidopsis, Populus, Vitis*, and *Oryza*) as a nSCG [22]. We have chosen this region as an example to test the utility of a nSCG for low-level phylogenies in comparison to eight widely studied chloroplast markers [20,21]. A single copy of this gene was identified in large EST sets from multiple basal angiosperms [22], making it a suitable candidate for our basal angiosperm test group *Tildenia*. The *agt1 *region was amplified, sequenced, and aligned for 62 accessions covering 33 out of the 59 species of *Peperomia *subgenus *Tildenia *resulting in a dataset of 2088 characters (Additional File 1: Taxa used in the present study, Additional File 2: Sequence statistics for the coding and non-coding regions for all markers in this study calculated with SeqState). A dataset with an identical sampling was generated for the *trnK *intron, the *matK *gene and the *trnK-psbA *spacer resulting in an aligned dataset of 3049 characters (sequences obtained in part from [51]. To allow further exploration of the utility of the *agt1*, we added additional chloroplast markers for a sub-sample of 26 representative accessions. These markers comprised the *psbA-trnH *spacer plus short parts of flanking genes (353 bp), as well as the *ndhF-rpl32-trnL *gene region, consisting of a partial sequence of the *ndhF *gene (204 bp), the *ndhF-rpl32 *spacer (620 bp), the *rpl32 *gene (158 bp) and partial sequence of the *rpl32-trnL *spacer (1048 bp) (Additional File 2: Sequence statistics for the coding and non-coding regions for all markers in this study calculated with SeqState). The *ndhF-rpl32 *spacer, *rpl32 *gene and *rpl32-trnL *spacer were co-amplified and yielded an aligned dataset of 2030 bp. All markers are among the most variable loci for phylogenetic studies according to Shaw *et al*. [20,21].

An important feature of *agt1 *in *Peperomia *is that PCR amplicons typically were resolved as a single sharp target sized band on agarose gels and, for most samples, could be sequenced directly without cloning. Only a few (roughly 10%) accessions required a cloning step. In those cases, a single band was detected in the agarose gel, but sequencing revealed short (1 to 10 bp) indels.

### *Structure and characterization of *agt1

**Figure 1:**
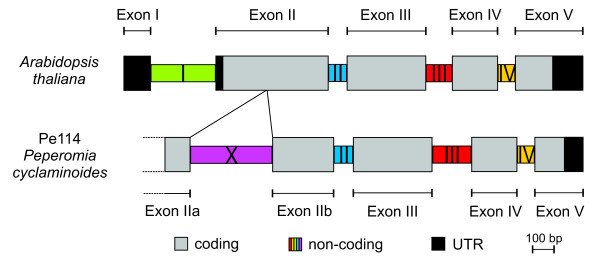
***The Alanine:Glyoxylate-Aminotransferase (*agt1*) gene model in *Arabidopsis thaliana *and in *Peperomia cyclaminoides**. The gene model for the *agt1 *gene in *P. cyclaminoides *Pe114 was derived from both genomic and cDNA sequences. Compared with the gene model in *Arabidopsis thaliana*, *Peperomia cyclaminoides *has an additional intron (intron X) that is 380 bp long and located in exon II. This intron can be found among all sequenced *Peperomia *accessions and ranges in length from 211-390 bp. We also checked its presence in a number of sequenced plant genomes available in Phytozome (v.7.0); the intron is absent only from *Arabidopsis *and grasses. Since it can be found in papaya, we suggest two independent losses of this intron: within the diversification of both Poaceae (or possibly a larger monocot group) and Brassicales.

In *Peperomia*, the *agt1 *gene comprises five coding regions (exons) separated by four intronic sections with a total length of 1542 to 1827 bp. In comparison to *agt1 *in *Arabidopsis thaliana*, the *Peperomia *gene contains an additional intron, which is located in the second exon and has a length of 211-390 bp (Figure 1). This intron is also present in the genomic sequences of *Populus trichocarpa, Medicago truncatula, Solanum lycopersicon, Oryza sativa, and Carica papaya*, as well as in all *Peperomia *species generated in the present study. Hence, it can be inferred that it was lost during the diversification of Brassicales. We named this additional non-coding section that splits exon II into two parts, intron X, and the two resulting exons that derive from exon II were named exon IIa and exon IIb (Figure 1). In the *Peperomia *accessions we studied, the *agt1 *exons are highly conserved in length and lack any indels. In contrast, intron X was the most variable in length among the respective four introns, showing a length range of 552-739 bp.

### *Variability of *agt1 *versus the chloroplast regions*

**Figure 2:**
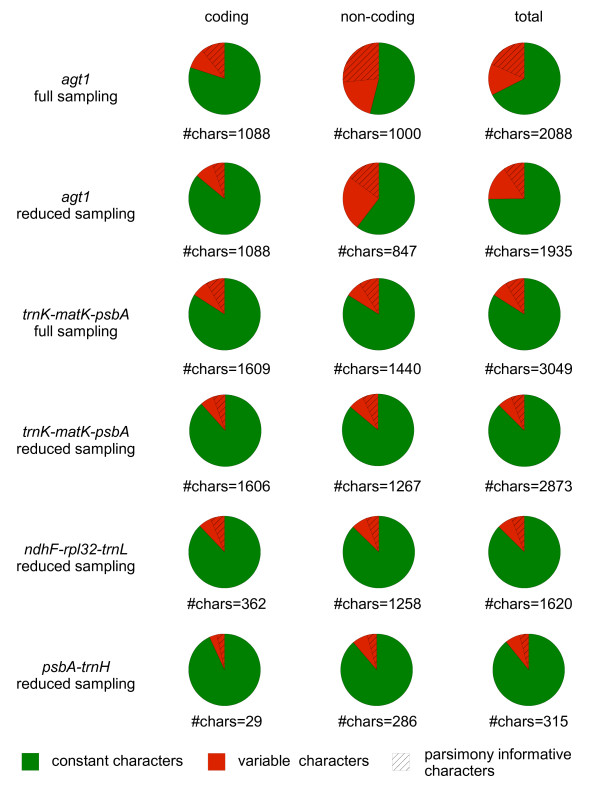
**Characteristics of utilized markers**. Portions of constant (green), variable (red) and parsimony informative characters (dashed) for coding and non-coding parts of all markers used in this study. Chloroplast markers suggested by Shaw *et al*. [19,20] for species level were combined into the longer regions that we co-amplified with single primer pairs. Full sampling comprises 62 accessions. Reduced sampling is a dataset that contains a sub-sampling of 26 selected accessions from the full sampling. The percentage of variability of the chloroplast markers is very similar between regions and among coding and non-coding portions at the taxonomic level in this study. In contrast, the nuclear gene has highly variable introns, which yields a total variability that is two to three times greater than any of the chloroplast markers.

To characterize the markers in more detail, several variability parameters were estimated for both coding and non-coding regions and for the full as well as the reduced sampling datasets (Figure 2, Additional File 2: Sequence statistics for the coding and non-coding regions for all markers in this study calculated with SeqState). In the *agt1 *full sampling dataset, introns and exons are roughly equally represented (introns 1000 bp, exons 1088 bp, see Additional File 2: Sequence statistics for the coding and non-coding regions for all markers in this study calculated with SeqState). Coding parts possess 20% variable characters (VC) and 11% parsimony informative characters (PIC), whereas non-coding parts contain 46% VC and 27% PIC. Thus, the content of PIC varies largely among different sections of the gene and in non-coding parts it is twice as high as in coding parts. In total, one fifth of the characters in the *agt1 *gene are parsimony informative.

The *trnK-matK-psbA *region does not vary significantly among coding and non-coding regions on this taxonomic level (Additional File 2: Sequence statistics for the coding and non-coding regions for all markers in this study calculated with SeqState). Compared to the *agt1 *with the identical sampling this region shows less than half of the ratio of VC and PIC (Additional File 2: Sequence statistics for the coding and non-coding regions for all markers in this study calculated with SeqState). Thus, the *agt1 *is much more effective in yielding PIC per sequenced bp than the widely applied *trnK-matK-psbA *region. Furthermore, the nuclear gene segments provide different amounts of variability, which make them useful for many taxonomic levels.

A similar pattern is visible in the reduced sampling of all utilized markers (Additional File 2: Sequence statistics for the coding and non-coding regions for all markers in this study calculated with SeqState). In total, the percentages of both variable sites (25%) and PIC (15%) of *agt1 *are much higher than those of the chloroplast markers. However, it has to be noted that the VC percentage of the reduced sampling is 7% lower than that of the full sampling and that there are only half of the PIC in the reduced dataset. In contrast, the *trnK-matK-psbA *does not possess large differences between reduced and full sampling datasets. The decrease of VC in the reduced sampling suggests that the potential of *agt1 *is not exhausted and deeper sampling would likely increase available variability and thus phylogenetic resolution.

The variabilities of the different gene regions are graphically displayed in Figure 2. It is remarkable that the chloroplast markers are very similar in their overall fractions of variable sites (Figure 2). Thus, the number of PIC is directly proportional to the number of sequenced bp and thus on this phylogenetic level marker selection does not seem to be crucial in terms of phylogenetic content. Furthermore, non-coding regions of all chloroplast markers are just as informative as non-coding regions on this phylogenetic level. To look for potential saturation of the markers, the retention index (RI) was calculated with and without indel-coded length mutations. Generally, for the chloroplast markers the RI does not differ between the datasets that include indels and those without indels (Table 1). The RI values of both *trnK-matK-psbA *datasets (full sampling including indels: RI = 0.873, reduced sampling including indels: RI = 0.828) as well as *ndhF-rps32-trnL *dataset (reduced sampling including indels RI = 0.836) indicate low homoplasy. The homoplasy of *agt1 *full sampling (including indels, RI = 0.802) is little homoplastic as well. Only the RI values of *psbA-trnH *(reduced sampling including indels RI = 0.697) and *agt1 *reduced sampling datasets (including indels: RI = 0.658) datasets are a little lower, which suggests that they are more homoplastic and likely closer to saturation. Considering the RI, both *trn*K-*mat*K-*psb*A and *ndh*F-*rpl*32-*trn*L can be regarded as little homoplastic. The RI of the different *agt1 *datasets differs and is lower in the reduced sampling. Consequently, a smaller sampling is more homoplastic than a denser sampling with the same breadth. This fact is congruent with the phylogenies of the *agt1 *gene, resulting in less resolution and support in the reduced dataset. This leads to the conclusion that a high variability as detected for the *agt1 *gene requires a dense sampling to keep the risk for artifacts low.

**Table 1:** Statistics based on a Maximum Parsimony Ratchet analysis showing the retention index as a measure of homoplasy.

		total characters (with indels)	Trees found (with indels)	Steps (with indels)	RI (with indels)
*agt1 *full sampling	coding	1088 (1088)	1253 (N/A)	405 (N/A)	0.827 (N/A)
	non-coding	1009 (1192)	1721 (1539)	978 (1205)	0.799 (0.794)
	total	2097 (2280)	232 (362)	1395 (1622)	0.802 (0.798)
*agt1 *reduced sampling	coding	1088 (1088)	206 (N/A)	260 (N/A)	0.742 (N/A)
	non-coding	856 (971)	323 (55)	613 (746)	0.636 (0.628)
	total	1944 (2059)	50 (64)	876 (1010)	0.667 (0.658)
*matK-trnK-psbA *full sampling	coding	1609 (1621)	158 (172)	448 (461)	0.882 (0.883)
	non-coding	1475 (1554)	39 (85)	422 (518)	0.879 (0.877)
	total	3085 (3174)	1007 (1356)	881 (990)	0.874 (0.873)
*matK-trnK-psbA *reduced sampling	coding	1606 (1614)	18 (15)	303 (312)	0.840 (0.383)
	non-coding	1310 (1361)	9 (28)	287 (343)	0.837 (0.831)
	total	2916 (2974)	12 (21)	587 (651)	0.832 (0.828)
*ndhF-rpl32-trnL *reduced sampling	coding	362 (367)	2 (2)	58 (63)	0.962 (0.964)
	non-coding	1668 (1783)	10 (8)	246 (382)	0.822 (0.823)
	total	2030 (2150)	1 (65)	305 (448)	0.847 (0.836)
*psbA-trnH *reduced sampling	coding	29 (29)	1 (N/A)	2 (N/A)	1.000 (N/A)
	non-coding	324 (370)	810 (28)	46 (100)	0.727 (0.714)
	total	353 (399)	1044 (38)	49 (103)	0.696 (0.700)

To summarize, a quarter of the total *agt1 *dataset is parsimony informative, while having a low homoplasy level at the same time. All calculations were based on nucleotide substitutions only (indels not included), as RIs of all data sets revealed this to be less homoplastic than the data sets containing indels (Table 1).

### Phylogenetic output

**Figure 3:**
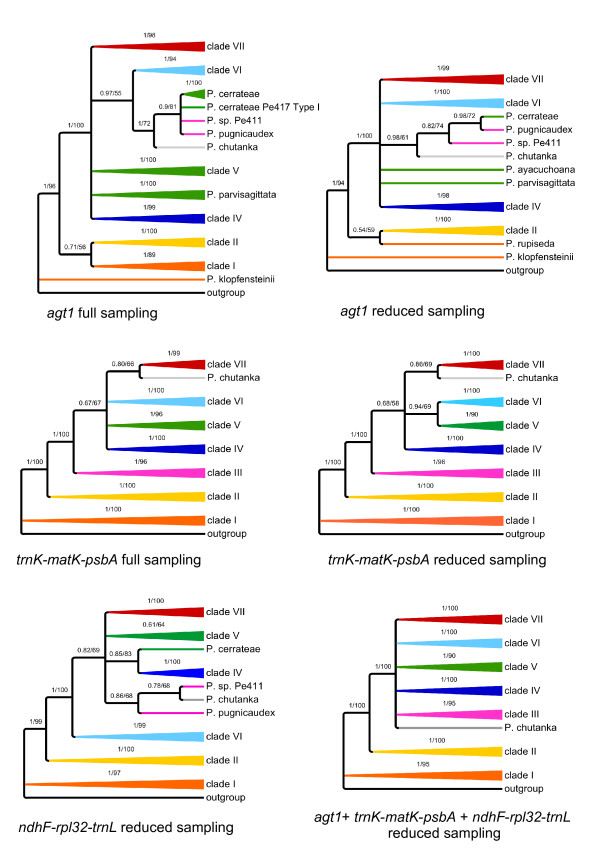
**Schematic summary of topologies**. Summarized topologies are based on Bayesian inference. Numbers above branches are posterior probabilities (PPs) from Bayesian inference (BI) (left) and ML bootstrap values (right). *Agt1 *and *trnK-matK-psbA *are shown for both full and reduced sampling, and *ndhF-rpl32-trnH *for reduced sampling. Clades were named based on the topology obtained from the *trnK-matK-psbA *region. Full sampling reflects a 63 accession dataset, while reduced sampling comprises 26 accessions. The chloroplast regions do not provide congruent signal. From all these trees, the nuclear gene provides the best resolution with the full sampling. In this tree, an additional clade is formed, consisting of *P. chutanka*, *P. pugnicaudex *and *P. cerrateae*. Clade VI is sister to this new clade. These (statistically supported) relationships cannot be observed in any of the trees based on chloroplast markers.

To obtain insights into *agt1*'s phylogenetic performance in terms of topology, resolution and support, we performed identical phylogenetic analyses with identical sampling and a reduced sub-sampling for all markers (Figure 3). For those analyses, Maximum Likelihood (ML) and Bayesian Inference (BI) were used. For simplicity, the clades have been named with numbers based on the study of Symmank et al. [51].

By looking at the trees derived from different markers, here summarized at the clade level (Figure 3), it is obvious that *agt1 *and the chloroplast gene trees result in different topologies. Although the majority of clades are recovered as monophyletic among all gene trees, a few clades are unresolved in the *agt1 *tree (Figure 3).

All phylogenetic trees obtained from the different markers show species from clade I and II as the first diverging branches followed by a polytomy formed by the remaining clades. The nuclear trees all show four clades out of seven being monophyletic and statistically supported (clade II, IV, VI and VII) (Figure 3). Clade I is polyphyletic in the *agt1 *tree, but monophyletic with any of the chloroplast markers. A new clade is formed in the *agt1 *phylogeny, consisting of *P. cerrateae*, *P*. sp. Pe411, *P. pugnicaudex*, and *P. chutanka*. Clade VI is sister to this clade, which is supported with 0.97 Posterior Probability (PP) in the full sampling. Thus, clades III and V are not monophyletic in the nuclear-based tree or in the tree resulting from *ndhF-rps32-trnL*. In the *trnK-matK-psbA *tree, *P. cerrateae *was part of clade V, *P*. sp. Pe411 and *P. pugnicaudex *formed clade III and the position of *P. chutanka *could not be resolved. In summary, *agt1 *yields a deeper resolution compared to any individual or combined chloroplast dataset by forming a new clade that has clade VI as a sister. Sampling density does not seem to influence the phylogenies obtained from the chloroplast markers as much as it affects the nuclear trees. Denser sampling seems to improve resolution with *agt1*. Statistical evaluation of phylogenetic signals between datasets was done using SH tests [58]. These tests reveal a significant conflict between the chloroplast markers and the nuclear *agt1 *gene, expressed by the rejection of all respective topology/dataset combinations (Additional File 3: Results from topology tests). The only exception to this pattern is the combination of the *ndhF-rpl32-trnL *topology and the *agt1 *dataset (p = 0.070). *The trnH-psbA *spacer was not used for this test due to insufficient phylogenetic resolution. In accordance with the result of Shaw *et al*. [20], this finding states the inappropriateness of this region for phylogenetic studies on a low taxonomic level.

To determine the source of conflict between the nuclear and chloroplast markers we tested 10 alternative hypotheses by changing the topologies of the reduced *agt1 *and *trnK-matK-psbA *datasets. In most cases a single clade was replaced according to its phylogenetic position in the opposed topology. Additionally, clades that appear polyphyletic in one topology were constrained to be monophyletic and placed at different positions. All alternative hypotheses resulted in significant rejection by the tested datasets. The alternative topologies and the SH-values of the accordant combinations are summarized in Additional File 3: Results from topology tests. The results indicate a general incongruence between the phylogenetic signal derived from the nuclear and chloroplast genome. The rejection of the alternative hypothesis clearly shows that the conflict is not caused by a single subset of species or clade, but is reflecting multiple inconsistencies between the nuclear versus the chloroplast gene regions at lower nodes of the tree. Such a pattern might be expected if an early hybridization and chloroplast capture event had occurred early in the evolution of the subgenus. Such an event can lead to incongruence between the histories of nuclear and plastid marker regions.

The combination of *agt1*, *trnK-matK-psbA*, *ndhF-rpl32-trnL*, and *trnH-psbA *data set (7243 total characters) yields a topology possessing all the monophyletic clades identified by *trnK-matK-psbA *with maximal support. In addition, *P. chutanka *is resolved in the combined analysis as sister to clade III (0.99 PP). A phylogeny of *trnH-psbA *alone did not yield any resolution (results not shown).

**Figure 4:**
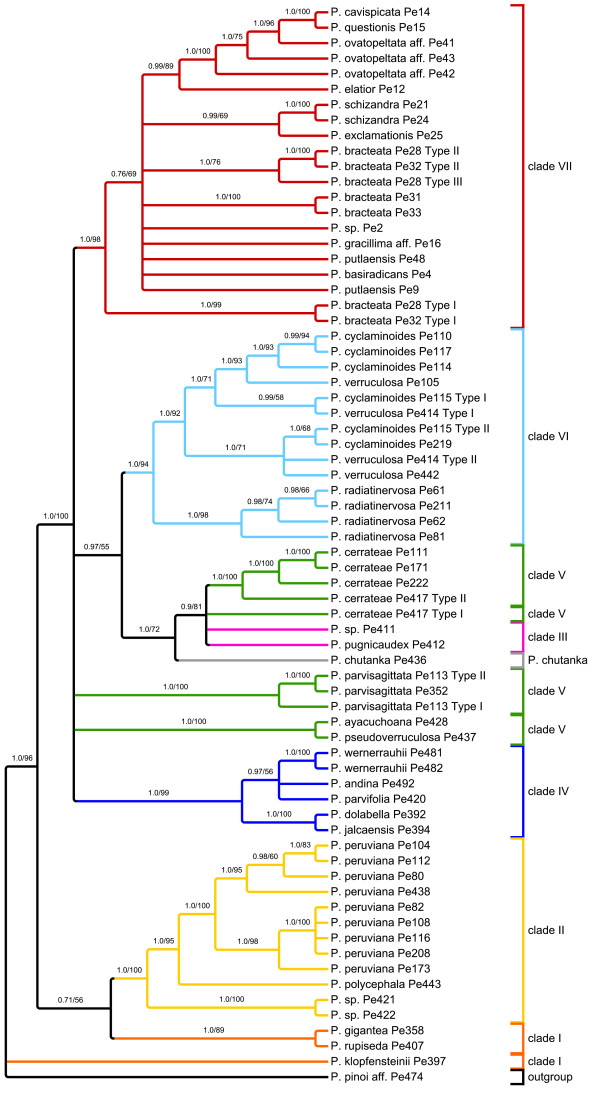
***Phylogenetic hypothesis for *Peperomia *subgenus *Tildenia *based on ML and BI analysis of *agt1**. This topology is derived from ML; nodes supported at < 50% in at least one of the two analyses were collapsed. Numbers above branches are PPs from BI (left) and ML bootstrap values (right).

**Figure 5:**
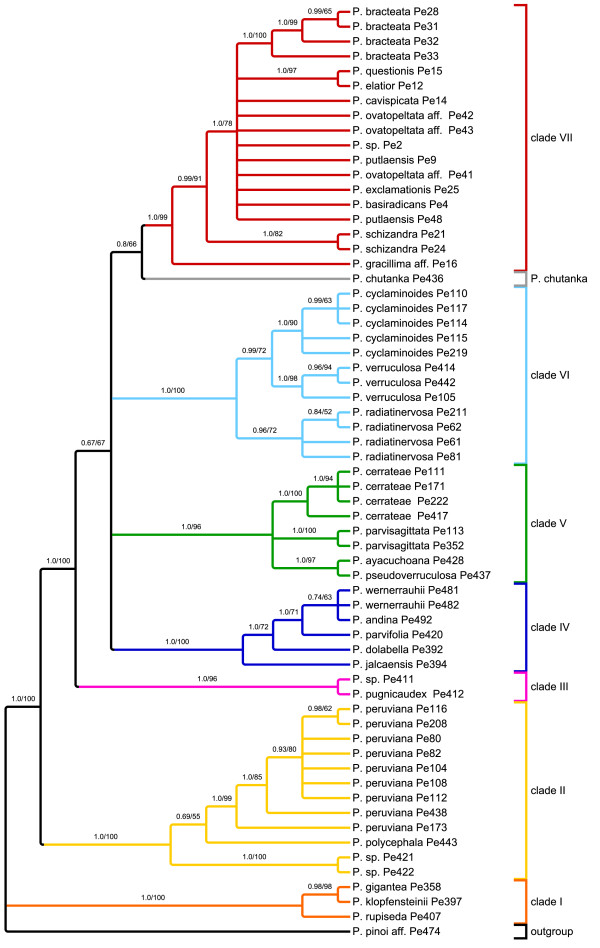
***Phylogenetic hypothesis for *Peperomia *subgenus *Tildenia *based on ML and BI analysis of *trnK-matK-psbA**. This topology is derived from ML; nodes supported at < 50% in at least one of the two analyses were collapsed. Numbers above branches are PPs from BI (left) and ML bootstrap values (right).

**Figure 6:**
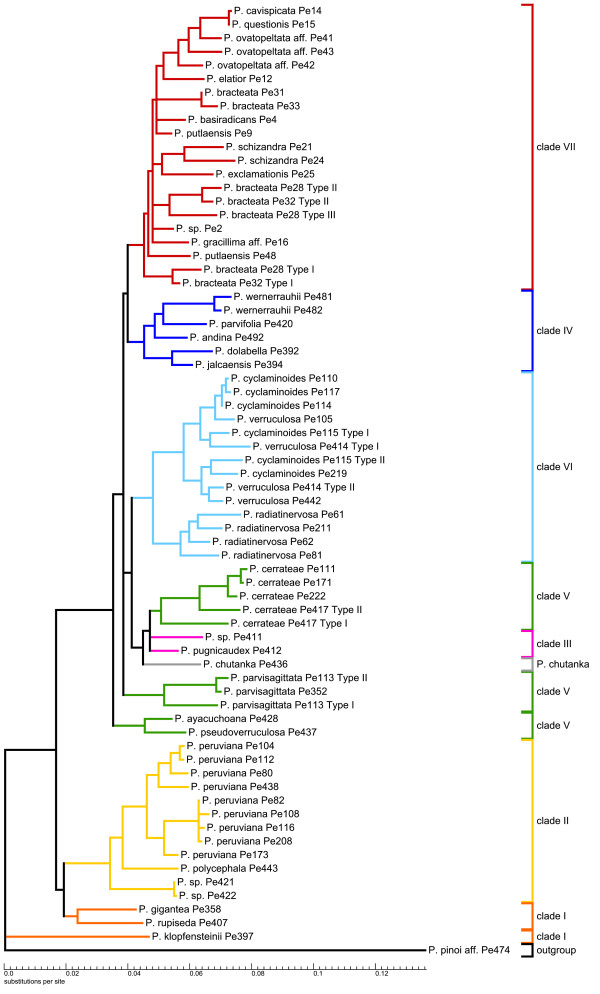
***Phylogram of *agt1 *based on Bayesian inference***. Phylogram obtained from the Bayesian inference of the *agt1 *data set with relative substitution rates using the GTR+G+I model and posterior probabilities plotted above the branches. This phylogenetic tree is summarized in Figure 3. Clades are colored according to Figure 3. In accessions that are represented in the tree by different 'types', multiple copies were detected that are not monophyletic. A closer look to the resolution within the clades shows highly supported relationships down to the population level.

**Figure 7:**
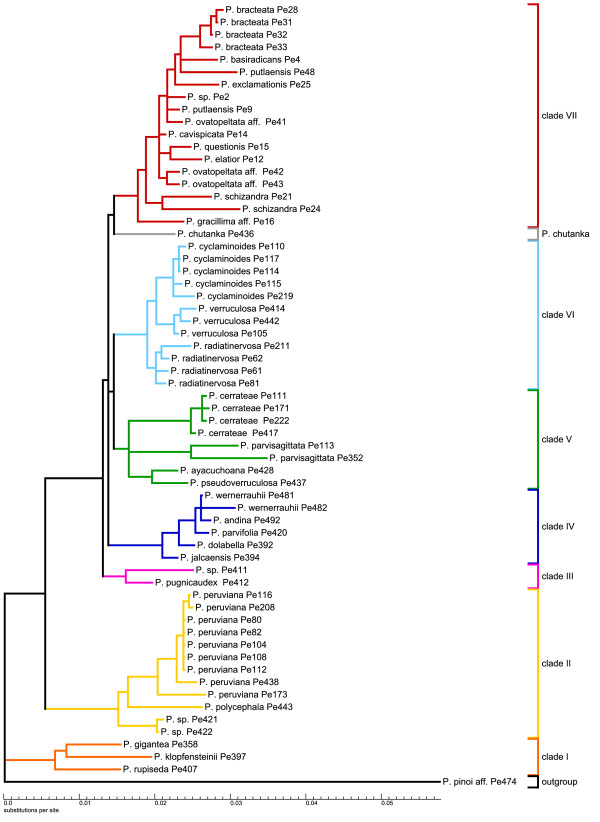
***Phylogram of the *trnK-matK-psbA region *based on Bayesian inference***. Phylogram from Bayesian inference of the *trnK-matK-psbA *data set with relative substitution rates using the GTR+G model and posterior probabilities plotted above the branches. This phylogenetic tree is summarized in Figure 3. Clades are colored according to Figure 3.

The resolution of the two full sampling phylogenies (Figure 4, Figure 5) was measured using the normalized consensus fork index (CFI), which divides the number of nodes found in a strict consensus tree by the number of possible nodes [59]. To increase the information value of this index with respect to the resolution capacity of the respective molecular markers, we consider the nodes that appear in the trees inferred from BI and ML, where nodes below 50% were collapsed (Figure 4 and 5). According to this, the *agt1 *yields a considerably higher total resolution (CFI = 0.74) than the *trnK-matK-psbA *region (CFI = 0.58). Comparison of the phylograms show that the substitution rate in the *agt1 *data set is about twice as great as in the *trnK-matK-psbA *dataset (BI, Figure 6 and 7).

The phylograms also suggest that a sudden and fast radiation occurred that gave rise to clades III-VII. This radiation has been linked directly to the formation of new habitats during the uplift of the inner Andean valleys in Peru and Bolivia [51]. Generally, the *agt1 *resolution is much higher within the clades and on population level, which is very obvious for *P. peruviana*, *P. cerrateae*, *and P. radiadinervosa *as well as within clade IV and VI (Figure 4). Furthermore, the *agt1 *phylogeny suggests that *P. cyclaminoides *and *P. verruculosa *are not monophyletic and *P. bracteata *is polyphyletic on the *agt1 *tree. Clade VII shows better resolution of the deeper branches with the chloroplast dataset (Figure 5), whereas the nuclear gene resolves relationships better in shallower parts of the tree. In the *trnK-matK-psb*A phylogenetic tree, *P. gracillima *aff. is the first branch, followed by *P. schizandra *as sister to the rest of clade VII. The nuclear gene, however, reveals a well-resolved sub-clade comprising *P. elatior *as the first branch, followed by *P. ovatopeltata *aff., *P. questionis *and *P. cavispicata*. Furthermore, in the nuclear phylogeny, *P. exclamationis *is resolved as sister to *P. schizandra*.

### *Single Copy vs. Multicopy status of *agt1

As described above, approximately 90% of the PCR amplicons could be directly sequenced from PCR product; only 10% required cloning to obtain distinct sequences. Multiple copies were detected in at least one accession from every clade except clade I and III. Putative pseudogenes, e.g. ones with frameshift mutations, were removed from the analysis. A resulting phylogeny, which contained all detected copies excluding putative pseudogenes, and which did not comprise sequence data downstream from intron III, revealed that the copies of eight accessions are monophyletic, and thus do not influence the final analysis (Additional File 4: Phylogram of *agt1 *based on BI of entire dataset including cloned sequences). Therefore, a cloned copy representing the particular accession could have been randomly chosen for the main phylogeny. However, *agt1 *copies sequenced from four of the accessions are not monophyletic. These copies cluster together with copies from other accessions of the respective species, indicating duplication during radiation of the respective species (*P. cerrateae *Pe417, *P. parvisagittata *Pe113, *P. schizandra *Pe24 and *P. wernerrauhii *Pe481) (Additional File 4: Phylogram of *agt1 *based on BI analysis of the entire dataset including cloned sequences). The only accessions for which the situation seems to be more complex are *P. bracteata *Pe28 and Pe32, as well as *P. cyclaminoides *Pe115 and *P. verruculosa *Pe414. Both cases show a similar pattern: copies of a single accession are not monophyletic, but are sister to their orthologous copy from other accessions. Thus, cloned sequences of such an accession are likely to be paralogous.

## Discussion

### Rapidly radiating genera and single copy nuclear genes

Rapidly radiating taxa and species-rich groups have been, and remain, challenging for high resolution phylogenetic analyses with widely used chloroplast markers [e.g. [60-62]].

Independent of the applied marker, most of these groups tend to share a well-resolved base with several long branches followed by several clades branching from a polytomy with rather short branches. This could indicate that some lineages within species-rich groups radiated over a comparatively short time span, possibly as a result of the availability of new habitat or some other opportunity. In the case of *Tildenia*, this branching pattern can be seen in the phylograms (Figure 6 and 7) and has been linked to geological events during the uplift of the Andes as well as the climatic oscillation during the period of diversification in the mid-Miocene between 15-10 MYA [51]. As we had expected, the *agt1 *gene resolves short and deep branches much better in *Tildenia*, since substitution rates for the non-coding parts are much greater than for coding or for non-coding chloroplast markers. Shaw et al. [21] concluded that non-coding chloroplast regions are the most useful for resolving relationships of closely related species, but we found non-coding parts of all chloroplast markers employed in the present study to be very similar in terms of variability compared to the coding sections of the chloroplast genome at this very low taxonomic level. In contrast, non-coding parts of nuclear genes show a three-fold increased variability compared to non-coding chloroplast markers.

Another reason why species rich genera are not as well studied as other groups is certainly because they are much more difficult to sample thoroughly and require both extensive field and herbarium work. This is the case for *Peperomia *where highly reduced flower morphology makes correct species identification a significant challenge [53]. The study by Wanke et al. [52] was the first to apply molecular markers to *Peperomia*. Currently, *Tildenia *is the best-studied subgenus of *Peperomia*, since there were many new species from the Andes and Mexico described recently [[55,56] respectively] and an extensive phylogeny was combined with a molecular dating approach to detect biogeographical migration patterns [51]. Our current study is primarily focused on the phylogenetic performance of *agt1 *compared to chloroplast markers, and thus, we applied a broad taxon sampling rather than several nSCG markers with a less dense sampling. For this reason, only limited conclusions can be drawn concerning the origin of the multiple copies as well as incongruent topologies of nuclear versus chloroplast trees. However, obtaining different signals from the nuclear gene in comparison to various chloroplast regions highlights how important it is to apply independent markers. In fact, many cases of incongruence between chloroplast and nuclear marker based topologies can be found in the literature [e.g. [63]]. However, the potential sources of novel clades in the nuclear gene tree seem to be very complex. There are completely different relationships formed on an interspecific level, deriving from many different clades in the *trnK-matK-psbA *tree. *Peperomia cerrateae*, *P. parvisagittata*, *P. ayacuchoana *and *P. pseudoverruculosa *formed a clade in the chloroplast-based tree. In the nuclear-based tree however, the species themselves are still monophyletic, but the relationships among species are poorly resolved. The simplest explanation for this pattern is that the *trnK-matK-psbA *tree lacks resolution at this level. This is because in the *ndhF-rpl32-trnL *tree a similar tendency is visible, in which *P. cerrateae*, *P. parvisagittata*, *P. ayacuchoana *and *P. pseudoverruculosa *are not a monophyletic clade and that *P. chutanka *might be related to clade III with *P. pugnicaudex*.

In the nuclear-based phylogenetic tree some cases are found where duplicated, paraphyletic copies of *agt1 *occur in a particular accession. An example for this is clade VI, which is formed of *P. radiatinervosa*, *P. cyclaminoides *and *P. verruculosa*. Since the four copies from *P. radiatinervosa *form a monophyletic clade, the duplication event could, for example, be due to alloploidy between the two other taxa. Another scenario that would explain this pattern is a duplication event that occurred after the split from *P. radiatinervosa*, but prior to the diversification of *P. cyclaminoides *and *P. verruculosa*. Under the latter scenario, duplicated copies in *P. radiatinervosa *and the other species evolved independently and are still retained in the respective accessions.

A second case of multiple *agt1 *copies in a species involves *P. bracteata*. *Agt1 *copies from this species form three different clades within clade VII: in two clades the different copies of Pe28 and Pe32 cluster together, whereas Pe31 and Pe33 lack duplicates. Incomplete lineage sorting could be an explanation for this pattern, since it is not shared with other species in this clade.

Our results confirm the conclusion that it is essential to use several unlinked markers for phylogenetic reconstruction to improve phylogenetic resolution, and to identify and limit gene tree artifacts, which can be caused by hybridization, polyploidy, introgression or lineage sorting [e.g. [50]]. However, to refine our understanding of the reasons for the observed conflicts between *agt1 *and chloroplast regions, additional nuclear loci would be required. The suite of AVPO SSCG loci identified by Duarte et al. [22] provide numerous candidates for further analysis.

### To be or not to be single copy

The starting point for the present study was a *Tildenia *phylogeny based on several chloroplast markers. In order to obtain a better resolution in *Tildenia*, in particular below the species level, we investigated the phylogenetic performance of the non-coding parts of one of the APVO SSCG [22]. Among the large collection of 965 SSCGs identified in *Arabidopsis*, *Populus*, *Vitis*, and *Oryza*, several were selected for further analysis of public EST datasets, and a trial analysis of Brassicaceae was performed using direct sequencing and phylogenetic analysis of a dozen of the genes using RT-PCR (cDNA) amplicons [22]. In both analyses, results were generally consistent with a single gene being detected in the expressed gene sets across diverse angiosperms, though duplicates were indicated in some species, particularly in recent polyploid taxa [49]. Thus, we reasoned that genes from among this collection were good candidates for further study in *Peperomia*, a basal angiosperm without prior nuclear gene sequence data. By amplifying and sequencing through intronic as well as exonic regions using primers grounded in conserved exons, a large collection of highly variable sites was obtained that were especially valuable for phylogenetic at and below the species level.

An advantage of the approach developed here is that it should be possible to take any angiosperm group of interest and quickly identify whether SSCG are likely to work well for species level phylogenetics. The only major requirement is a pair of unambiguous amplification primers that will work for the particular group of interest. In some cases, primers from Duarte *et al*. [22] or this study may serve as amplification or sequencing primers. However, additional steps can be taken to enhance the chances of success in any given study. Publicly available EST data can easily be queried to extract sequences from closely related taxa, perform alignments with genome and coding regions of the sequenced species obtained from the PlantTribes database [39], and design primers that have an increased likelihood of success. Given the large number of 965 APVO SSCG [22], successful primer pairs should be identified for at least a handful of genes for any angiosperm group. In our study, after one initial round of primer design for *Tildenia *and the first sequencing run, *agt1 *was one of six genes that immediately yielded good results. This gene did prove to be low copy (rather than single copy) in some *Tildenia *accessions, which is not unexpected given that all angiosperms have a history of ancient polyploidy and both polyploidy and gene duplication is a frequent process in plants [64-66].

The rapidly increasing number of sequenced plant genomes allows the identification of genes that are present in single copy across increasingly many species. The recently updated PlantTribes database 2.0 [67] provides a publicly available tool that can be used for identifying such genes. If the database is queried (at moderate stringency, 3.0) for Tribes containing a single gene in each of the seven angiosperm genomes (*Arabidopsis*, *Populus*, *Vitis*, *Oryza*, *Sorghum*, *Medicago *and *Carica*), this number decreases to 223 genes and if three non-angiosperm plant genomes are added (*Selaginella*, *Physcomitrella*, and *Chlamydomonas*), 36 genes pass the criteria. We see that as new genomes are added to this approach, there is a decrease in the number of shared single copy genes. However, this set of genes is much more refined and thus may be more likely to be single copy throughout plants in general. In the case of *agt1*, beside the APVO species, it occurs in a single copy in *Sorghum bicolor *and *Carica papaya*, but there are six copies in the *Medicago truncatula *(Version MT3.5) genome. In the three non-angiosperm plant genomes, two annotated copies can be identified in *Selaginella moellendorfii*, three in *Chlamydomonas reinhardtii*, and five in *Physcomitrella patens*.

The approach we develop here differs from another recent method to identify orthologous nuclear markers within a particular plant family or other relatively specific lineage [e.g. [44]]. In a group that already has multiple genome-scale datasets, such as the large angiosperm families Asteraceae, Solanaceae, Poaceae, and Fabaceae, genomes and EST datasets can be leveraged to identify genes that exist in single copy across the range of sampled taxa. In this case, larger numbers of shared single copy genes may be obtained within a family, but the marker set will include many genes that are multicopy in other lineages.

Even if a number of 'nearly universal' nSCGs are eventually identified in angiosperms, universal amplification and sequencing primers that can be used in an arbitrarily chosen lineage are perhaps less likely to exist than for chloroplast genes due to the relatively rapid underlying substitution rates for nuclear genes. Nucleoribosomal markers, such as the intergenic transcribed spacer region (ITS), can often be amplified and sequenced with widely conserved primers because it is flanked by highly conserved nucleoribosomal gene sequence [68] and do provide an independent estimate of phylogenetic history in comparison to chloroplast DNA. However, because ITS regions form a large multicopy "family" where copies are (often incompletely) homogenized through concerted evolutionary processes, assessment of orthology in nuclear ITS sequences is very complex, even when multiple copies are cloned and sequenced [23,24]. We found that nSCG amplification and primer design requires a little more lab effort compared to chloroplast markers, but on the other hand, unlinked loci from different genomic compartments are essential in order to reconstruct and fully interpret phylogenetic relationships [69]. It is comparably easy to generate a data set of several chloroplast markers, but those results need to be complemented and compared to unlinked nuclear markers.

## Conclusions

Considering topology, variability and homoplasy, we can conclude that *agt1 *is highly valuable for phylogenetics at a low taxonomic level such as species level and below. Beyond that, *agt1 *was useful for identifying further evolutionary biological processes such as recent gene duplication. When combined with additional nuclear genes, numerous concurrent duplications would be indicative of recent polyploidization in a species or even in particular populations. In combination with uniparentally inherited loci, biparentally inherited nSCGs can also provide evidence for hybridization and potentially for organelle capture. The nSCG applied in the present study provides regions with different quantitative levels of variability deriving from coding and non-coding parts of different length. While the percentage of PIC of the coding sections does not vary significantly among the chloroplast and nuclear markers, the non-coding sections of the *agt1 *in *Peperomia *is three times higher than the most rapidly evolving chloroplast regions.

## Methods

### Sampling Strategy

Based on Symmank *et al*. [51], Mathieu *et al*. [55] and Samain *et al*. [56], we sampled a phylogenetically representative subset (29 out of 59 species) of *Peperomia *subgenus *Tildenia *(Additional File 1: Taxa used in the present study). As outgroup taxon, *Peperomia pinoi *aff. Pe474, closely related to this subgenus, was chosen [52,53]. For a comprehensive approach to compare the *agt1 *gene with the *trnK *intron, *matK *gene and the *trnK*-*psbA *spacer region, 70 accessions including the outgroup species were sampled. In addition, the *agt1 *gene was compared to two additional chloroplast gene clusters containing both introns and spacers (*ndhF*-*rpl32*-*trnL *and *psbA*-*trnH*). A restricted set of 26 accessions including outgroup was utilized for this.

### Marker selection

The starting point for the present study were 959 genes identified by Duarte *et al*. [22], that are shared in single copy in the annotated genomes of *Arabidopsis thaliana*, *Populus trichocarpa*, *Vitis vinifera *and *Oryza sativa*. These authors used a high throughput comparative proteomic approach to identify genes that occur in single copy in all of the four genomes. This comprehensive approach suggests candidate markers for any angiosperm of interest, because the genes captured this way are present in phylogenetically diverse angiosperms and thus potentially suitable useful as common phylogenetic markers in varied taxa.

Starting from 13 potential nSCGs compared by Duarte *et al*. [22], ESTs (Expressed Sequence Tags) obtained from the MAGIC database from the Ancestral Angiosperm Genome Project [70] were screened for homologues in other representatives of basal angiosperm lineages. The sequences of these 13 loci were extracted from all available plant genomes in order to check the variation of total length as well as the length and number of introns. Our decision to use the *agt1 *gene was made based on the number of introns and the positive impact that introns would have on the expected variability for that gene. Comparison of EST data and further characterization was done using PlantTribes [39,67] and TIGR Plant Transcript Assemblies [71,72]. Initial primer sets were designed for this particular region using both Sanger and 454 (Roche) EST data from other basal angiosperm lineages (*Amborella trichopoda*, *Nuphar advena*, *Liriodendron tulipifera*, *Persea americana, Aristolochia fimbriata*) to subsequently amplify and sequence the gene region in a RT-PCR approach for *Piper nigrum *and *Peperomia prostrata *(4474-390F: ACCAGGGAGGAACCATCTCTTTG; 4474-1530R: TTYTTCARMCCCCATGCTTC). These sequences were used to build primers that are more specific for Piperaceae to amplify and sequence *Peperomia *accessions. A single primer was designed in a highly conserved 5' region of the gene (Pe-4474-1800F: TTCTTTGAYTGGAATGACTACTTGA) which was applied in a 3'-RACE (3' RACE System for Rapid Amplification of cDNA Ends, Invitrogen Corporation) for *Peperomia cyclaminoides *Pe114. Based on the resulting sequence, primers at the outermost end of the gene were generated and then used for a broad set of *Peperomia *accessions (Additional File 5: Primers used for amplification and sequencing of both the nuclear and chloroplast markers applied in this study).

### DNA isolation, RNA isolation, cloning, amplification, RT-PCR and sequencing

DNA was isolated from silica dried material using the CTAB method. Most of the sequences of the *trnK-matK-psbA *gene region are obtained from Symmank *et al*. [51]. Amplification of additional *trnK-matK-psbA *sequences as well as other chloroplast sequences followed the procedure described in Symmank *et al*. [51]. Primers used for amplification and sequencing of the chloroplast markers are listed in Additional File 5: Primers used for amplification and sequencing of both the nuclear and chloroplast markers applied in this study. For the nuclear marker, both 25 μl and 50 μl reactions were run containing between 0.5 and 4 μl DNA template (100 to 200 ng) for a 50 μl reaction. A 'master mix' for a 50 μl reaction contained 8 μl dNTP (Roth, 1.25 mM each), 5 μl red Taq-buffer for high yields (PeqLab), 1.5 μl MgCl_2 _(25 mM), 1 μl of each primer (50 pmol/μl) and 0.5 μl of Taq DNA polymerase (PeqLab). Water was added to obtain a total reaction volume of 50 μl. For amplification of the *agt1 *we modified a standard PCR-program for low concentration of target DNA. The program started with an initial denaturation step at 94°C for 2 min, followed by 45 cycles of denaturation at 96°C for 45 sec, annealing at 51°C for 30 sec and elongation at 72°C for 90 sec, and a final elongation 72°C for 7 min. Annealing temperature was adjusted to the melting point of the primers. PCR was run on a T3 Thermocycler (Biometra). After gel electrophoresis through a 1.2% agarose gel, the PCR products were purified using a gel extraction kit (Macherey&Nagel). Some of the PCR products required an additional cloning step. In those cases the T/A Cloning Kit (Genaxxon BioScience) was applied following the manufacturer's protocol. A ligation reaction was set up with 2-4 μl PCR-product, 1 μl of each ligase buffer, ligase and vector and filled up with water to a total reaction volume of 10 μl. Three to nine clones were arbitrarily chosen and directly amplified via colony PCR using M13 primers under the following conditions: initial denaturation at 95°C for 2 min, denaturation at 95°C for 1 min, annealing at 55°C for 1 min, elongation at 72°C for 1.5 min and a final elongation at 72°C for 10 min. This PCR was run with 36 cycles and the products were directly sequenced after purification (Macherey&Nagel).

RNA was isolated using peqGold Plant RNA Kit (PeqLab) following the manufacturer's instructions. A 3' RACE kit (Invitrogen Life Science) was used following the manufacturer's protocol. For RT-PCR the Access RT-PCR System (Promega) was used following the manufacturer's instructions.

Direct sequencing was conducted either using a Beckman Coulter CEQ DTCS Quick Start Kit (Beckman Coulter) with the CEQ 8000 sequencer or using Macrogen's sequencing service (Macrogen Inc., Korea). Sequences were edited and aligned manually using PhyDE [73]. Regions of uncertain homology (e.g. long monobase repeats) were excluded from all subsequent analyses.

Due to the length of the *agt1 *gene, DNA was not amplified in a single reaction but in three parts with substantial overlap (50 to 150 bp) to ensure efficient amplification. The ESTs and cDNA sequences of basal angiosperms mentioned above, which were available to generate initial amplification primers, did not cover the entire region. The 3'-ends of the ESTs usually reached exon IV, making a RACE approach necessary to include full-length gene information including intron IV and exon V. The middle section comprising only the short intron II (between exon IIb and exon III) yielded sequences without any length mutations, whereas either the first or the third part or both sections required a cloning step for several accessions and were treated individually. Very few PCR amplicons (approximately 10%) revealed differences in length among amplified products. The three sections of those accessions had to be assembled together to gain a full sequence. The assemblage yielded up to three different 'types' of copies and the fragments were sorted phylogenetically. This method allowed us to avoid artifacts due to incorrect sequence combinations.

However, most of the detected multicopy accessions were monophyletic in the phylogenetic tree and thus, a copy could be chosen at random for the further analysis. For the few accessions that appeared polyphyletic in our analyses, a copy of each 'type' was left in the dataset and was distinguished by 'type I' and 'type II'. All major clades of subgenus *Tildenia *contained at least one accession that required cloning steps; thus multiple copies are not unique to a particular lineage.

### Phylogenetic analyses

Several mostly small regions of uncertain sequence homology (hotspots) had to be excluded from the different data matrices (Additional File 6: Hotspots excluded, due to ambiguous homology assessments). All alignments are available from TreeBASE.

Indel matrices were calculated using the "simple indel coding" approach (SIC) [74]. This indel matrix was generated automatically by the indel coding tool of SeqState [75]. Substitution models for Bayesian inference (BI) were determined using jModelTest [76]. For the *agt1 *dataset the general time reversible model of nucleotide substitution and site-specific rate categories following a gamma distribution (GTR+I+Γ) was assigned as the best fitting model considering the Akaike information criterion (AIC). For the three chloroplast datasets, GTR+Γ was the best fitting model. Bayesian MCMC inferences were performed with MrBayes v3.1 [77] using the substitution models mentioned above.

The BI was applied with four Markov chains running simultaneously for 4 million generations, saving trees every 100 generations. The burn-in was individually set for each analysis between 5% and 20% after determining stationarity of each run with Tracer v1.5 [78]. At least ten runs were assembled to generate the consensus trees and posterior probabilities for each individual analysis. Maximum Likelihood as implemented in RAxML Version 7.2.7.a [79] using the rapid bootstrap algorithm was used in order to increase the number of bootstrap replicates to 1,000.

The degree of homoplasy of each dataset both with and without indels was assessed on a Maximum Parsimony (MP) tree that was obtained using a parsimony ratchet approach. Command files for MP analyses were created using PRAP [80] and executed in PAUP*4b10 [81]. Topologies were obtained with the heuristic search strategy and 10 random addition cycles of 200 iterations each with a 25% upweighting of the characters in the iterations. For compiling and drawing all trees TreeGraph2 [82] was employed.

Sequence statistics for specified regions of each marker were obtained utilizing SeqState [75]. The Shimodaira-Hasegawa (SH) test [58] was performed in PAUP*4b10 [81] with full optimization to evaluate the topologies obtained by the different genetic markers against each other. The SH test simultaneously compensates for *a posteriori *hypotheses of multiple alternative topologies by adjusting the expected difference in log-likelihood values. Topology tests were performed on the *agt1 *dataset containing one randomly selected single copy to comply with the congruence of sampling. In case of conflict between two markers (*p ≤ *0.05) the test was repeated with a manually modified topology to determine the conflict. In the process, one hypothesis (topology) was stepwise adjusted to the phylogenetic results of the conflicting marker to evaluate the effects of single clade position changes.

## Authors' contributions

JN: laboratory work for the nSCG dataset, cloning, phylogenetic analyses and drafted the paper. LS: collected samples, contributed the cpDNA datasets, carried out phylogenetic analyses, statistical tests, and helped draft the paper. MSS: collected samples, helped with the sampling design (including identification and nomenclature) and proofread the paper. KFM: supplied funding for the study, edited the draft version, helped with the statistical analysis. CN: supplied funding for the study and lab space for parts of the study, and proofread the paper. CWD: helped draft the manuscript, supplied funding and lab space for parts of the study, contributed in the discussion in various phases, analysis of EST datasets, and proofread the paper. SW: designed and supervised the study, supplied funding for the study, performed RT-PCR, primer design, and sample collection, and helped draft the paper. All authors read and approved the final manuscript.

## Supplementary Material

Additional file 1 **Taxa used in the present study**. Excel file listing the species used in this study including voucher information and GenBank accession numbers (provided upon final acceptance of manuscript). For detailed information of origin and collection sites see [49]. Click here for file

Additional file 2 **Sequence statistics for the coding and non-coding regions for all markers in this study calculated with SeqState**. This table provides information about the general characteristics of the coding, and non-coding parts of the study sequences for different sampling densities. Click here for file

Additional file 3 **Results from topology tests**. Excel file showing Shimodaira-Hasegawa (SH) test results of different topology/dataset combinations. Asterisk indicates a significant rejection (p ≤ 0.05) of the respective combination. Click here for file

Additional file 4 ***Phylogram of *agt1 *based on a Bayesian inference of full dataset containing cloned sequences***. Phylogram from the Bayesian inference of the *agt1 *data set with relative substitution rates using the GTR+G+I model and posterior probabilities plotted above the branches. Clades are colored according to Figure 3. The applied data set comprises part I and part II of the nuclear gene including all cloned sequences. Click here for file

Additional file 5 **Primers used for amplification and sequencing of both the nuclear and chloroplast markers applied in this study**. Excel file listing primer sequences of the respective gene region that were used for both PCR and sequencing. Click here for file

Additional file 6 **Hotspots excluded, due to ambiguous homology assessments**. Regions of uncertain sequence homology (hotspots) excluded from the different datasets. Positions are given equivalent to files downloadable from TreeBase. Click here for file
