# Supplementary figures and images for: Chasing the hare - Evaluating the phylogenetic utility of a nuclear single copy gene region at and below species level within the species rich group Peperomia (Piperaceae)
Source: BMC Evol Biol. 2011 Dec 12;11:357. doi: 10.1186/1471-2148-11-357 (PMC3252395; doi:10.1186/1471-2148-11-357)

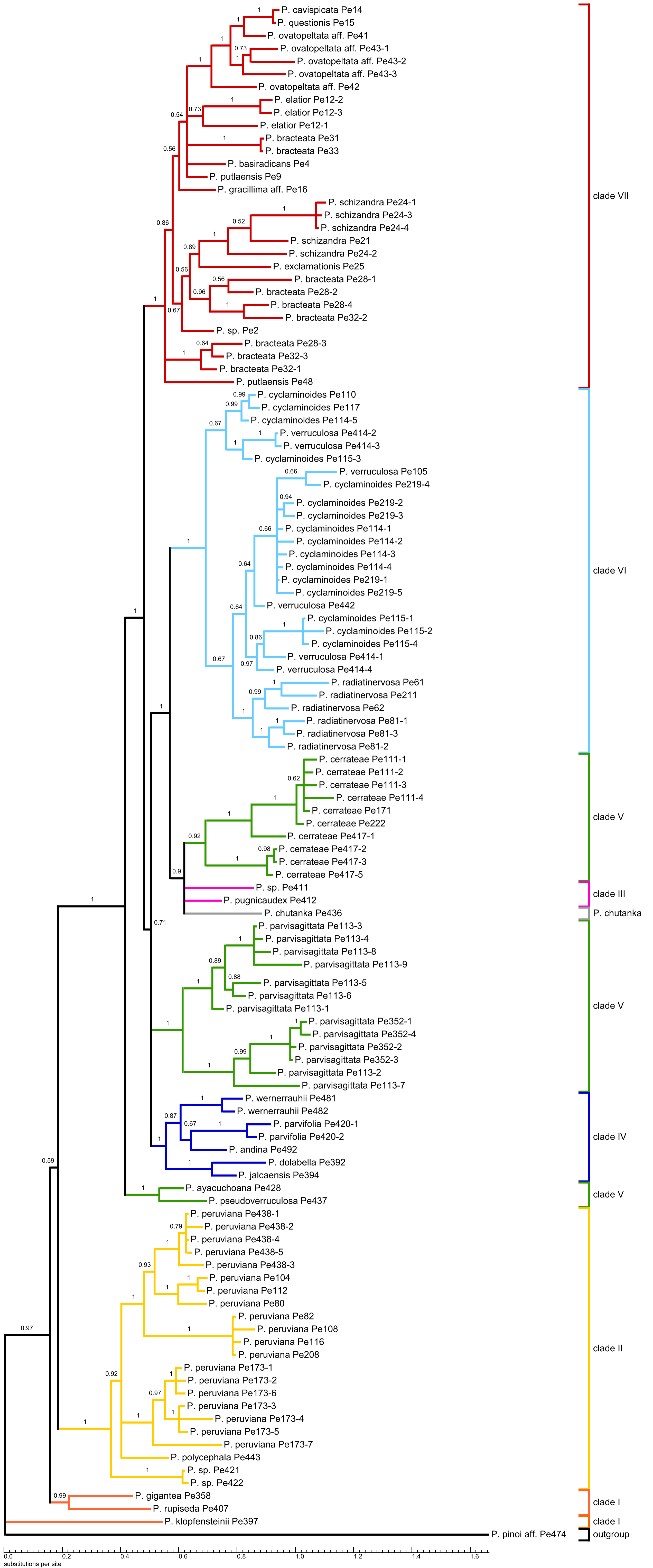

Supplement: Additional file 4 — Phylogram of agt1 based on a Bayesian inference of full dataset containing cloned sequences. Phylogram from the Bayesian inference of the agt1 data set with relative substitution rates using the GTR+G+I model and posterior probabilities plotted above the branches. Clades are colored according to Figure 3. The applied data set comprises part I and part II of the nuclear gene including all cloned sequences. [file 1471-2148-11-357-S4.PDF]
